# Simultaneous splicing of multiple DNA fragments in one PCR reaction

**DOI:** 10.1186/1480-9222-15-9

**Published:** 2013-09-09

**Authors:** Wei-Gui Luo, Hui-Zhen Liu, Wan-Huang Lin, Mohammed Humayun Kabir, Yi Su

**Affiliations:** 1 Hunan Provincial Key Laboratory of Phytohormones, Hunan Agricultural University, Changsha 410128, China

**Keywords:** Simultaneous splicing, Multiple DNA fragments, Overlap extension PCR

## Abstract

**Background:**

Rapid and simultaneous splicing of multiple DNA fragments is frequently required in many recombinant DNA projects. However, former overlap extension PCRs, the most common methods for splicing DNA fragments, are not really simultaneous fusing of multiple DNA fragments.

**Results:**

We performed an optimized method which allowed simultaneous splicing of multiple DNA fragments in one PCR reaction. Shorter outermost primers were prior mixed with other PCR components at the same time. A sequential thermo cycling program was adopted for overlap extension reaction and amplification of spliced DNA. Annealing temperature was relatively higher in the overlap extension reaction stage than in the fused DNA amplification. Finally we successfully harvested target PCR products deriving from fusion of two to seven DNA fragments after 5–10 cycles for overlap extension reaction and then 30 cycles for fused DNA amplification.

**Conclusions:**

Our method provides more rapid, economical and handy approach to accurately splice multiple DNA fragments. We believe that our simultaneous splicing overlap extension PCR can be used to fuse more than seven DNA fragments as long as the DNA polymerase can match.

## Background

Fusion of two or more DNA fragments is frequently required in many recombinant DNA projects but conventional restriction enzyme cloning and adapters cannot work well in many cases. Overlap extension PCR [1-3], USER fusion [4-6] and some other developed techniques [7-9] all can be used to fuse DNA fragments. Original overlap extension PCR, known as OE-PCR, was employed to splice two fragments precisely and quickly without the use of restriction enzymes [1-3]. Some modified OE-PCR methods can be applied in many areas, such as long-length DNA fusion [10-12], long-length gene cloning [13-15], gene mutation [16-18] and DNA circularization [19]. Although some researchers have successfully assembled up to four DNA fragments simultaneously [10], multiple DNA splicing remains a challenge and no evidences show that multiple DNA fragments fusion based on OE-PCR is a universal approach or not. Here we modified OE-PCR, referred to simultaneous splicing overlap extension PCR (SSOE-PCR), which was allowed to simultaneously assemble seven DNA fragments in one PCR reaction without the use of ligation based on restriction enzymes. We believe that our SSOE-PCR can be used to splice more than seven DNA fragments as long as the DNA polymerase can match. In this new method, the outermost primers were added with other PCR components at the same time before beginning overlap extension reaction, but higher annealing temperature was setup during overlap extension stage. Moreover, it allows directly running the next programs with lower annealing temperature in the same reaction solution after overlap extension phase. Our method will be not only more faster and flexible than regular restriction enzyme cloning, but also more rapid, economical and handy than other OE-PCR protocols [8,10,17,18,20,21].

## Results and discussion

**Figure 1:**
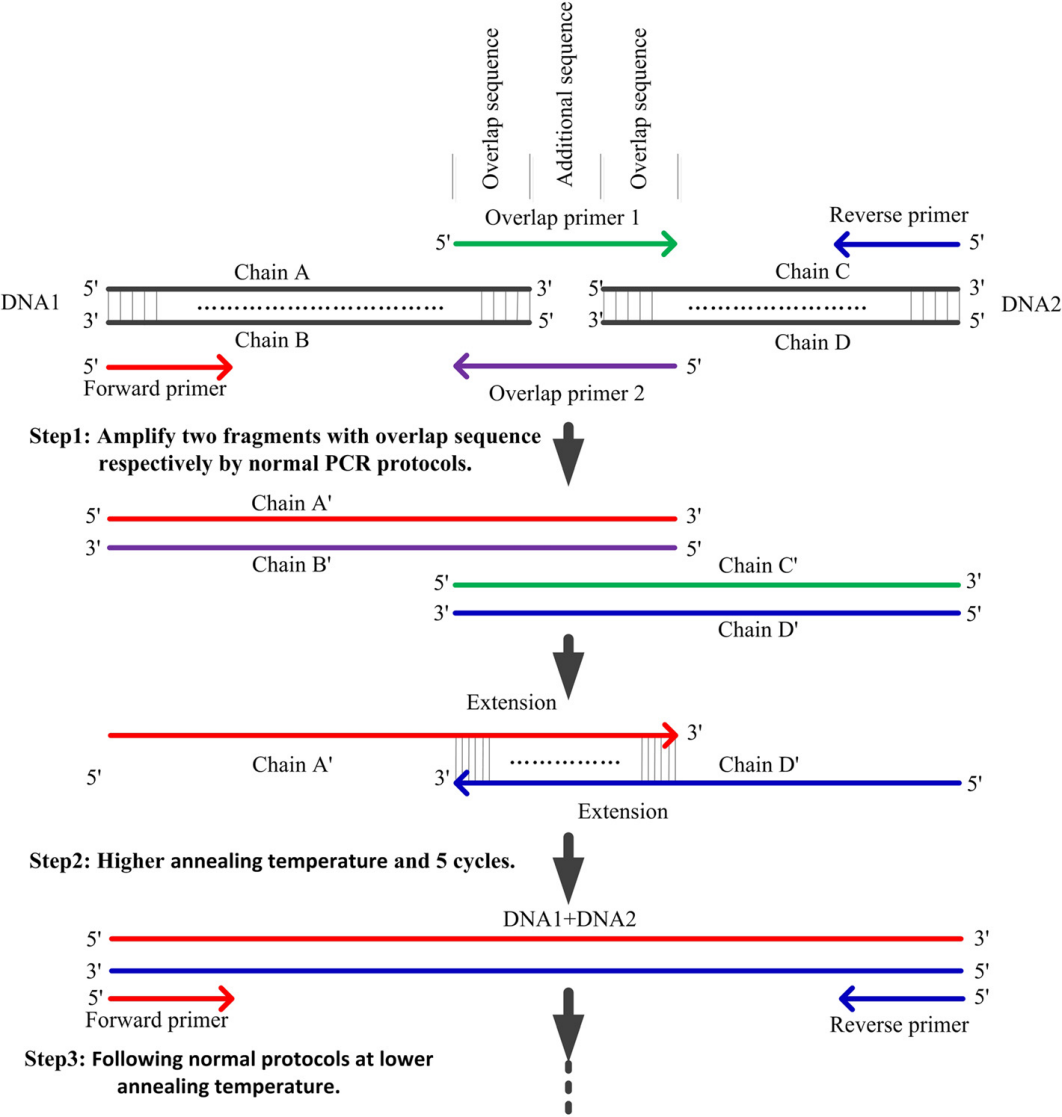
**Principles of our simultaneous splicing overlap extension PCR (SSOE-PCR).** Step 1, two fragments with overlap sequences are amplified by normal PCR after choosing better outmost primers (Forward primer and Reverse primer) and overlap primers. Step 2, a program of five cycles is employed at higher annealing temperature after correctly mixing DNA polymerase buffer, dNTPs, few of the two fragments, outermost primers and Pfu DNA polymerase as well as adding water to certain volume. Some fused DNAs (DNA1 plus DNA2) will be synthetized. Step 3, a continuous program of 30 cycles following step 2 is edited and running at lower annealing temperature through normal PCR protocols.

Overlap extension PCR was initially employed for fusion of two or three DNA fragments. The classical overlap extension PCR method generally consists of two steps and two separated reaction mixtures i.e. (1) In the overlap extension phase the outermost primers were no need and annealing temperature was relatively lower than the next step (2) In the fused DNA amplifying phase, a new reaction mixture with the outermost primers was made and a new program was run at higher annealing temperature. The overlap extension products merely acted as templates in second step. We combined these two steps of reactions in our SSOE-PCR: i.e. one PCR solution with all the components including the outermost primers was made and one continuous cycling program was employed at higher annealing temperature in overlap extension stage but lower in the fused DNA amplifying stage (Figure 1 and Table 1).

**Table 1:** Comparison between classical overlap extension PCR (OE-PCR) and our simultaneous splicing overlap extension PCR (SSOE-PCR)

**Method**	**Overlap extension phase**			**Fused DNA amplifying phase**			**Number of reaction system**	**Thermo cycling programs**
	**Outermost primers**	**Annealing temperature**	**Function of outermost primers**	**Outermost primers**	**Annealing temperature**	**Function of outermost primers**		
Classical OE-PCR	No need	Lower	_	Need	Higher	Yes	One by one	Separate
SSOE-PCR	Need	Higher	No	Need	Lower	Yes	One	Continuous

**Figure 2:**
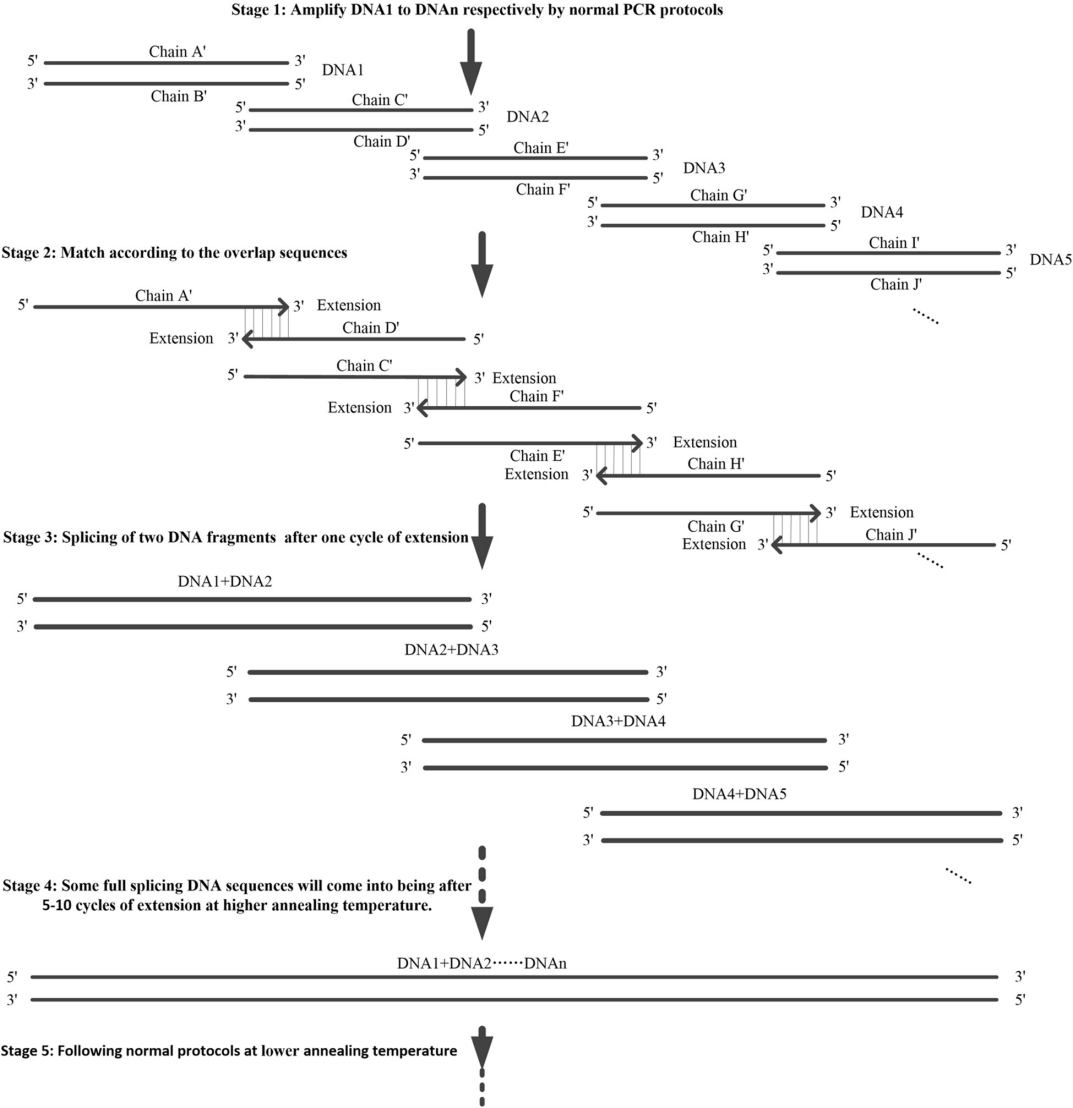
**Hypothetical principle of multiple DNA fragments fusion by overlap extension PCR.** Stage 1: designing primers and amplifying DNA1 to DNAn with overlap sequences respectively through normal PCR; Stage 2: One chain of a fragment matched with another one. Stage 3: Single chain matching with another one extended along 5′ to 3′ at higher annealing temperature and then a series of double splicing DNAs might come into being after one cycles. Step 4: One double splicing DNA could continuously match with another one and then extend, and so forth. Finally, some full multiple splicing DNAs would always be synthetized after more several cycles in the former condition. Stage 5: Amplifying full multiple fusion DNAs at lower annealing temperature through normal PCR.

Multiple DNA fragments can be simultaneously fused together in one reaction by SSOE-PCR. A hypothetical principle may explain how it works (Figure 2). First, DNA1 to DNAn with overlap sequences can be amplified respectively by normal PCR and programs (stage 1 in Figure 2). Second, all the PCR components including DNA1 to DNAn are mixed together and a single chain of one fragment will match with another one at properly higher annealing temperature, such as chain A′ belonging to DNA1 will match with chain D′ of DNA2 and so on (stage 2 in Figure 2). A series of double splicing products may be arisen after few cycles of extension and then longer splicing products after more cycles (stage 3 in Figure 2). Some fully spliced DNAs will always come into being after 5–10 cycles of extension (stage 4 in Figure 2). Finally, the outermost primers will exert their function in the next 30 cycles cycling program with lower annealing temperature and full length of multiple fuse DNA can be amplified (stage 5 in Figure 2). Similarity to double fusion is that outermost primers with other PCR components are mixed at the same time, and overlap extension reaction and full length fused DNA amplification reaction are working in one tube. The difference between double fusions and multiple fusions is that the multiple fusions may need more cycles.

**Figure 3:**
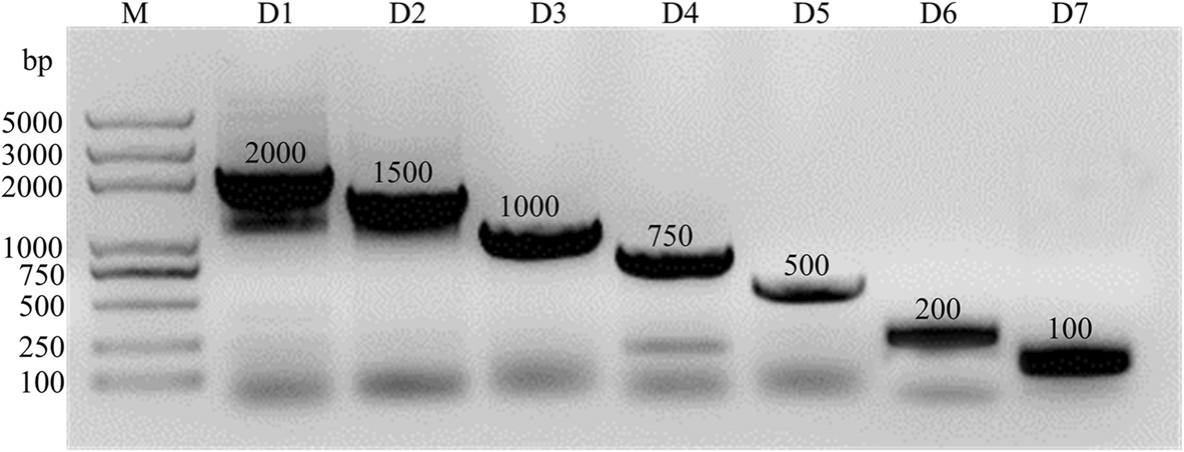
**PCR products of seven different fragments.** Lanes from left to right represented DNA molecular weight marker, DNA fragment D1 (2000 bp), D2 (1500 bp), D3 (1000 bp), D4 (750 bp), D5 (500 bp), D6 (200 bp) and D7 (100 bp).

Seven different lengths of DNA fragments have been amplified well through seven pairs of primers after 35 cycles. All the target lines showed high specificity in 1% agarose gel (Figure 3). Half of PCR products were purified by gel extraction kit and re-dissolved in 30 μl water. The recycling rate of gel extraction was about 85%. The results of alignments all perfectly matched with DNA sequences anticipated after sequencing PCR products (data not shown).

**Figure 4:**
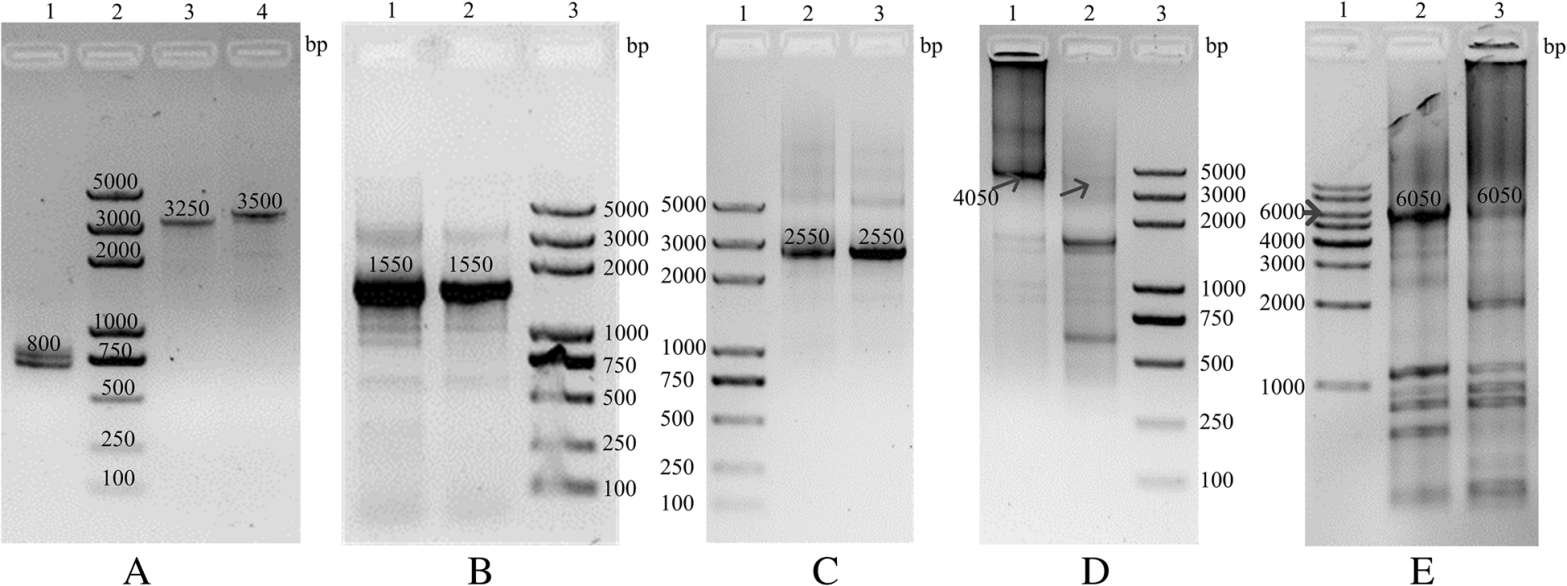
**Electrophoretic analysis of different number of DNA fragments fusions in 1% agarose gel. (A)** Two and three DNA fragments fusion: lane 1 (800 bp), D5:D6:D7 fusion; lane 2, DNA molecular weight marker; lane 3 (3250 bp), D2:D3:D4 fusion; lane 4 (3500 bp), D1:D2 fusion. **(B)** Four DNA fragments fusion (D4-D7): lane 1 (1550 bp), unpurified PCR products as templates; lane 2 (1550 bp), purified PCR products as templates; lane 3, DNA molecular weight marker. **(C)** Five DNA fragments fusion (D3-D7): lane 1, DNA molecular weight marker; lane 2 (2550 bp), unpurified PCR products as templates; lane 3 (2550 bp), purified PCR products as templates. **(D)** Six DNA fragments fusion (D2-D7): lane 1 (4050 bp, arrow), unpurified PCR products as templates; lane 2(4050 bp, arrow), purified PCR products as templates; lane 3, DNA molecular weight marker. **(E)** Seven DNA fragments fusion (D1-D7): lane 1, DNA molecular weight marker; lane 2 (6050 bp), unpurified PCR products as templates; lane 3(6050 bp), purified PCR products as templates.

According to the principles of SSOE-PCR (Figures 1 and 2), we successfully fused two and three DNA fragments by using our simultaneous splicing overlap extension PCR (Figure 4A). Then we tried to simultaneously fuse more than three DNA fragments to make sure the extensional applications of modified overlap extension PCR. All reaction components were mixed together according to Table 2 and the cycling program were followed according to the upper protocols. Interestingly, we have successfully amplified the multiple fused DNA whenever unpurified or purified PCR products were used as the templates (Figure 4B-E). Higher specificity candidate lines have been harvested in four and five fusions than in six and seven DNA fragments fusion. The results of six and seven DNA fragments fusion showed that use of unpurified PCR products as the templates were better.

**Table 2:** Components of PCR solution for splicing of different numbers of DNA fragments

^**1**^**Components**	**Two**	**Three**	**Three**	**Four**	**Five**	**Six**	**Seven**
	**Volume (μl)**						
Total	30	30	30	30	30	30	30
ddH_2_O	15.7	14.7	14.7	13.7	12.7	11.7	10.7
^2^Buffer	3	3	3	3	3	3	3
dNTPs	3	3	3	3	3	3	3
Primer1	3(D1F)	3(D5F)	3(D2F)	3(D4F)	3(D3F)	3(D2F)	3(D1F)
Primer2	3(D2R)	3(D7R)	3(D4R)	3(D7R)	3(D7R)	3(D7R)	3(D7R)
*Pfu*	0.3	0.3	0.3	0.3	0.3	0.3	0.3
D1	1	–	–	–	–	–	1
D2	1	–	1	–	–	1	1
D3	–	–	1	–	1	1	1
D4	–	–	1	1	1	1	1
D5	–	1	–	1	1	1	1
D6	–	1	–	1	1	1	1
D7	–	1	–	1	1	1	1

^1^The orders of components were not necessarily taken into account when making PCR reaction mixture.

^2^When using the unpurified DNA fragments as the templates we need subtract the amount of buffer in templates.

**Figure 5:**
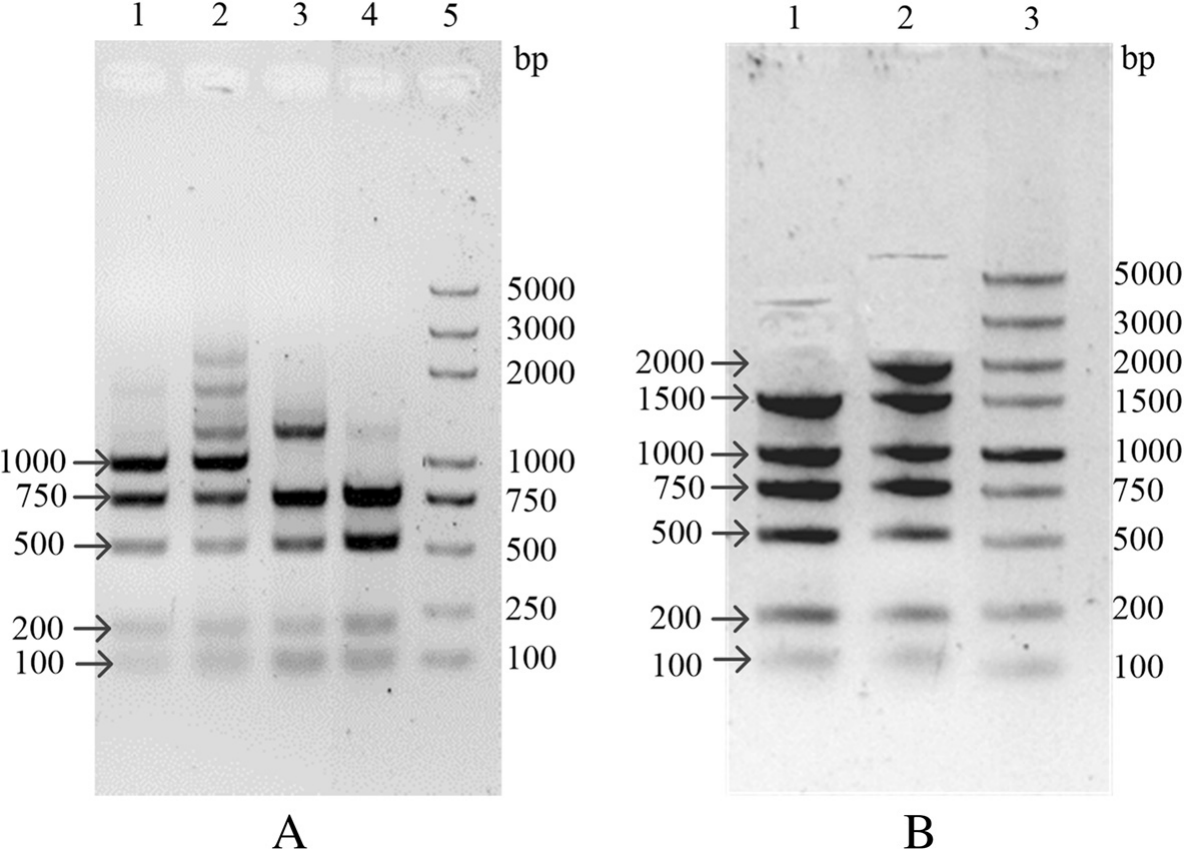
**Detecting the digestion product of fusion DNAs by *Eco *RI in 1% agarose gel. (A)** Lane 1: five fusion (D3-D7, 2550 bp) from lane 2 of Figure 2C; Lane 2: five fusion (D3-D7, 2550 bp) from lane 3 of Figure 4C; Lane 3: four fusion (D4-D7, 1550 bp) from lane 1 of Figure 2B; Lane 4: four fusion (D4-D7, 1550 bp) from lane 2 of Figure 4B; Lane 5: DNA molecular weight markers. Lines more than 1 kb indicated that the fused DNAs were not fully digested. **(B)** The PCR products were digested by *Eco*RI after purifying the target DNA (4050 bp in lane 1 of Figure 4D and 6050 bp in lane 2 of Figure 4E) by gel extraction kit and then amplifying them by PCR. Lane 1: six fusion (D2-D7); Lane 2: seven fusion (D1-D7); Lane 3: DNA molecular weight markers.

However, the recombinant candidate DNA fragments may be amplified from the leftover vector pGADT7 but not from the fused DNA since pGADT7 was used as the template for PCR reactions to generate all seven fragments. To dispel this doubt, the candidate DNAs were digested by *Eco*RI and then run in 1% agarose gel. The result showed that the anticipated bands were clear in gel (Figure 5). Therefore, it was verified that multiple DNA fragments have been successfully assembled.

The classical OE-PCRs generally contained three reactions: (1) Two different DNA fragments with overlap sequences were amplified through DNA polymerase (2) A PCR mixture with DNA polymerase buffer, Mg^2+^, dNTPs, two types of purified PCR products coming from the previous step and DNA polymerase except primers was made, and processed a 5–10 cycles cycling program at lower annealing temperature (3) Using the second PCR products (fully fused DNA) as the templates, amplification was done through the outermost primers (Table 3). Therefore, the classical OE-PCRs are suitable to fuse two or three DNA fragments, but not strictly simultaneous splicing.

**Table 3:** Name of the DNA fragments, the nucleotide sequence used for designing the PCR primers and product length

**DNA fragments name**	**Primer name**	**Primer sequences**	**Product length (bp)**
D1	D1F	5′gaattcACCCGGGTGGGCATCG3′	2000
	D1R	5′CCGGCTTTTCATATAgaattcGAATAGAGAAGCGTTCATGAC3′	
D2	D2F	5′GTCATGAACGCTTCTCTATTCgaattcTATATGAAAAGCCGG3′	1500
	D2R	5′GAAGATCCTTTTTGATgaattcAATCTCATGACCAAAATC3′	
D3	D3F	5′GATTTTGGTCATGAGATTgaattcATCAAAAAGGATCTTC3′	1000
	D3R	5′CCGCTCATGAGACAgaattcATAACCCTGATAAATG3′	
D4	D4F	5′CATTTATCAGGGTTATgaattcTGTCTCATGAGCGG3′	750
	D4R	5′GTAGAGGTCGAGTTTgaattcAGATGCAAGTTCAAGG3′	
D5	D5F	5′CCTTGAACTTGCATCTgaattcAAACTCGACCTCTAC3′	500
	D5R	5′GAATCTATACTTCTTgaattcTTTTGTTCTACAAAAATG3′	
D6	D6F	5′CATTTTTGTAGAACAAAAgaattcAAGAAGTATAGATTC3′	200
	D6R	5′GACCTGCAGGCATGCAgaattcGCTGCATTTTTACAG3′	
D7	D7F	5′CTGTAAAAATGCAGCgaattcTGCATGCCTGCAGGTC3′	100
	D7R	5′gaattcACCCTTATATTTGTC3′	

We combined the last two reactions, modified the cycling program and realized simultaneous splicing two or more DNA fragments. Therefore, we called this method simultaneous splicing overlap extension PCR (SSOE-PCR). The protocols of our SSOE-PCR are as follows: First, overlap primers containing two parts of sequences respectively matching with DNA1 and DNA2 must be designed and then two longer DNAs with overlap sequences are amplified by normal PCR program (Figure 1 step 1). Second, a five cycles cycling program is employed at higher annealing temperature decided by overlap primers′ Tm after correctly mixing all the components, including DNA polymerase buffer, dNTPs, few of the two types of PCR products coming from last step, outermost primers, *Pfu* DNA polymerase and certain volume of ddH_2_O (Figure 1 step 2). Third, a 30 cycles cycling program, keeping in step with earlier 5 cycles of overlap extension reaction, is working at lower annealing temperature decided by the outermost primers′ Tm and the spliced DNA will be amplified (Figure 1 step 3). In SSOE-PCR, the important modifications are designing shorter outermost primers (forward primer and reverse primer), mixing the outermost primer in step 2 (Figure 1), setting higher annealing temperature in overlap extension phase and running a continuous cycling program in one reaction mixture. In fact, the outermost primers could not match with DNA1 or DNA2 and affect PCR reaction in step 2 because the Tm value of overlap sequences was higher than the outermost primers.

According to the principles of our SSOE-PCR, splicing of two or more genes becomes very easy and it is a convenient tool when making constructions without restriction enzyme. Classical OE-PCR can be introduced to splice genes, make certain mutations and circulate linear DNA [16-19]. In this research, all the components including specific primers were mixed together in advance before the cycling staring programs of step 2 (Figure 1). That increased the stability of reaction system and made the protocol more easily operating.

Although OE-PCR becomes a general method in making construction as well as some researchers have already assembled four different linear DNAs through a modified OE-PCR [9], the process based on two separated reactions and wasn’t strictly simultaneous splicing. In this research, we tried to simultaneously splice more than two DNA fragments after mixing all the reaction components (following Table 2: DNA polymerase buffer with Mg^2+^, dNTPs, templates, primers and *Pfu* DNA polymerase) and successfully harvested the anticipated PCR products in one PCR reaction (Figure 2). To introduce multiple DNA fragments into a vector we need to use multiple restriction enzymes but sometimes enough restriction enzymes sites may not be available. Therefore, our SSOE-PCR will be a convenient and powerful tool in this regard.

Overlap extension PCR was described more than twenty years ago [1-3] and have been developed as a general tool for recombinant plasmid construction. As a powerful tool, several factors can affect the quantity and quality of PCR products such as primers, annealing temperature, template quality, ion environment and DNA polymerase. For successfully splicing multiple DNA fragments more attentions were given for primer designing, annealing temperature and templates selection in this research.

Designing somewhat shorter primers is helpful for handy operation. In general principle, the designing of primers needs to be in the range of 15–30 bases. In this experiment, the lower limit (15 or 16 bases, Table 3) was chosen for shortening the base quantity of overlap primers to control their melting temperature within the range of 68–70°C (accordingly, annealing temperature during long PCR is 65–68°C). Too long overlap primers bring about too high Tm and so annealing temperature set up in step 2 (Figure 1) may be not the best choice.

Setting higher annealing temperature is the key factor in the first several cycles of overlap extension reaction in SSOE-PCR. In this method, the length of overlap primers was almost double of the outermost primers (forward and reverse primers). Two heterogeneous single chains can match according to the overlap sequences but cannot match with the outermost primers at higher annealing temperature. Therefore forward and reverse primers, prior added to PCR reaction solution of step 2, don’t affect the overlap extension reaction in the first several cycles. We can directly setup the next cycling programs with lower annealing temperature in the phase of amplification of fused DNA fragments, but it is no need to make a new PCR reaction solution.

In fact, the DNA fragment with overlap sequences not only functions as template but also primer in the process of step 2 (Figure 1). The quantity and quality of heterogeneous DNA fragments may affect reaction. However, there is a novel phenomenon that the specificity of candidate line was unexpectedly better when using not purified PCR products as the template than purified by agarose gel, especially in six and seven DNA fragments fusion (Figure 4). Gel-purification of longer fragments made them not suitable for long multiple splicing but acceptable for short fusion under six fragments. The most likely reason is the damage to the template that results from exposure to ethidium bromide and UV light. Long PCR is ordinarily much more sensitive to the quality of the template [22]. Moreover, DNA is more stable in a buffer with pH above 7, but the DNAs repeated freezing and thawing or storage in distilled water are not fit as template for long PCR [10,22]. Unpurified PCR products can function as the excellent templates since DNA is of high stability in PCR buffer. At the same time, directly use of PCR products as the template is an economical way for less time and fewer chemicals consumption. If one does have to gel-purification of long PCR products, it is recommended that very low level of ethidium bromide should be used in agarose gels (not more than 0.15 mg/ml) and the gel never exposed to direct UV light [10].

## Conclusions

In conclusion, our protocols of simultaneous splicing overlap extension PCR have the following characteristics: (1) Designing shorter outermost primers with lower Tm (accordingly, setting lower annealing temperature) can make sure that the outermost primers have no function in the first several cycles of OE-PCR when they are simultaneously added into PCR reaction mixture together with other components (2) Setting higher annealing temperature can make the candidate splicing DNA extend fully in the first several cycles of OE-PCR and increase the specificity of final PCR products in the next cycles, but the outermost primers cannot match with one of DNA fragments in the higher annealing temperature condition. If the annealing temperature is installed according to the outermost primers or even lower following the former researches, the specificity will decrease dramatically and some shorter fragments will arise (data not shown) (3) Outermost primers are simultaneously mixed together with other components and a series of continuous programs are set up. It is a rapid, economical and handy approach since there is only one PCR reaction solution and a series of continuous programs when integrating overlap extension PCR and the next normal PCR. Other limiting factors such as the kind of DNA polymerase and the length of DNA fragments are attentively taken into account. Therefore, the high fidelity and efficiency DNA polymerase with a 3′-5′ proofreading activity, such as *Pfu* or *Phusion* DNA polymerase but not *Taq*[23], should be applied in splicing multiple DNA fragments (especially long fragments). In this research we have successfully fused seven DNA fragments but our team harvested highly specific candidate DNA sequence splicing from nine shorter fragments (60 bp-1 kb) when making a construction (not published). Therefore, our method is expected to become a universal approach in multiple genes fusion.

## Methods

### Primer designing for splicing of DNA fragments

We randomly chose seven DNA fragments (D1:2 kb, D2:1.5 kb, D3:1 kb, D4:750 bp, D5:500 bp, D6:200 bp and D7:100 bp) derived from a vector pGADT7. D1F and D1R were used to amplify fragment D1; D2F and D2R were used to amplify fragment D2. The rest could be deduced from this. Outermost primers D1F and D7R were designed with minimal requirement length of 16 and 15 bases respectively (Table 3). And lower melting temperature (Tm) of outermost primers allowed them working well at lower annealing temperature. One overlap primer (D1R to D7F in Table 3) generally contained two parts of sequences with more than a dozen bases coming from two different DNA fragments. For example, 5′CCGGCTTTTCATATA3′ in primer D1R derived from D1 could match with one chain of D1, but 5′GAATAGAGAAGCGTTCATGAC3′ in primers D1R derived from D2 and could match with one chain of D2; D1R is the reverse complement sequence of D2F. Vector pGADT7 contain unique *Eco*RI site and fragment D1 stars from this site. Moreover, restriction enzyme sites of *Eco*RI (small letters in Table 3) were employed at the ends of the seven fragments for digestion analysis to confirm the results. Fragments D1 to D7 were amplified by using pGADT7 as the template.

### Preparation of a series of different lengths of DNA fragments

Two tubes of 30 μl volume of reaction solution were prepared for every DNA fragment: 17.2 μl ddH_2_O, 3 μl 10 × *Pfu* DNA polymerase buffer with MgSO_4_ (Beijing TransGen Biotech Co. Ltd., China), 3 μl 2.5 mM dNTPs (Sigma, USA), 3 μl 5 μM forward primer, 3 μl 5 μM reverse primer, 0.5 μl 0.1 ng per milliliter of pGADT7 solution, 0.3 μl *Pfu* DNA Polymerase (500 bp/min, Beijing TransGen Biotech Co. Ltd., China). Cycling parameters: initial denaturation at 94°C for 4 min, denaturation at 94°C for 30 s, annealing at 54°C for 30 s, extension at 72°C following different times, 35 cycles, hold at 4°C. All PCR products were electrophoresed in 1% agarose gel. Half of PCR products were purified from agarose gel by using EasyPure™ Quik Gel Extraction kit (Beijing TransGen Biotech Co. Ltd., China).

### DNA fragment splicing

All components, including ddH_2_O, *Pfu* DNA polymerase buffer (containing Mg^2+^), dNTPs (2.5 mM), primers (5 μM), DNA fragments (above PCR products acting as templates) and *Pfu* DNA polymerase, were simultaneously added into a tube according to Table 2. Unpurified PCR products were directly used as the templates when splicing double or triple DNA fragments. Two triple fusions in Table 2 were merely different in the length of target products. At the same time, two tubes were employed to make reaction solutions to compare the simultaneous multiple fusion rates when using purified PCR products or unpurified as the templates. Generally, former researchers firstly setup 5–10 cycles of program for overlap extension without primers and secondly the PCR products were used as templates for the next 30 cycles of PCR. Here, only one continuous program was employed and cycling parameters were as follows: initial denaturation at 94°C for 4 min, denaturation at 94°C for 30 s, annealing at 60°C for 30 s, extension at 72°C (time following the longest fragment), 5–10 cycles for overlap extension; then denaturation at 94°C for 30 s, annealing at 54°C for 30 s, extension at 72°C (time following full length of fused DNA), 30 cycles for amplifying fully spliced DNA, finally hold at 4°C. PCR products were electrophoresed in 1% agarose gel.

## Competing interests

All authors declare that they have no competing interests.

## Authors’ contributions

All authors read and approved the final version of this manuscript.
